# The effect of citrate in cardiovascular system and clot circuit in critically ill patients requiring continuous renal replacement therapy

**DOI:** 10.1007/s10047-022-01329-0

**Published:** 2022-04-12

**Authors:** Thananda Trakarnvanich, Phatadon Sirivongrangson, Konlawij Trongtrakul, Nattachai Srisawat

**Affiliations:** 1 Faculty of Medicine, Vajira Hospital, Navamindradhiraj University, 681 Samsen Road, Dusit, Bangkok, 10300 Thailand; 2 Department of Medicine, Somdech Phra Pinklao Hospital, Bangkok, Thailand; 3 Faculty of Medicine, Pulmonary, Critical Care, and Allergy Division, Internal Medicine, Chiang Mai University, Chiang Mai, Thailand; 4 Excellence Center for Critical Care Nephrology, King Chulalongkorn Memorial Hospital, Bangkok, Thailand; 5 Critical Care Nephrology Research Unit, Chulalongkorn University, Bangkok, Thailand; 6 Academy of Science, Royal Society of Thailand, Bangkok, Thailand

**Keywords:** Acute kidney injury, CRRT, Clot circuit, Regional citrate anticoagulation, Heparin

## Abstract

We aimed to evaluate the impact of citrate on hemodynamic responses and secondary outcomes, including the filter life span, metabolic complications, and levels of inflammatory cytokines, in critically ill patients who required CRRT compared with those who underwent the heparin-free method. This prospective, multicenter, open-label randomized trial compared regional citrate anticoagulation (RCA) with a heparin-free protocol in severe acute kidney injury (AKI) patients who received continuous venovenous hemodiafiltration (CVVHDF) in the postdilution mode. We measured hemodynamic changes using the FloTrac Sensor/EV1000™ Clinical Platform at certain time points after starting CRRT (0, 6, 12, 24, 48, and 72 h.). The levels of inflammatory cytokines (IL-1β, IL-6, IL-8, IL-10 and TNF-ɑ) were measured on days 1 and 3. Forty-one patients were recruited and randomized into the heparin (*n* = 20) and citrate groups (*n* = 21). The cardiac performances were not significantly different between the 2 groups at any time point. The inflammatory cytokines declined similarly in both treatment arms. The maximum filter survival time was insignificantly longer in the RCA group than in the heparin-free group (44.64 ± 26.56 h. vs *p* = 0.693 in citrate and heparin free group). No serious side effects were observed for either treatment arm, even in the group of liver dysfunction patients. RCA did not affect hemodynamic changes during CRRT. Inflammatory cytokines decreased similarly in both treatment arms.The filter life span was longer in the citrate group. RCA is a valid alternative to traditional anticoagulation and results in stable hemodynamic parameters.

**Supplementary Information:**

The online version contains supplementary material available at 10.1007/s10047-022-01329-0.

## Introduction

Acute kidney injury (AKI) has a significant association with high mortality in critically ill patients and occurs in up to 50% in all critically ill patients in intensive care units (ICUs) [1]. Continuous renal replacement therapy (CRRT) has been recommended and widely applied for the management of severe AKI in critically ill patients with hemodynamic instability according to various guidelines [2–4]. CRRT is often preferred over intermittent hemodialysis (IHD) in the ICU, because it is associated with less hemodynamic instability and suitable for patients with at risk of increase intracranial pressure due to the slower fluid removal and the absence of fluid shifts induced by rapid solute removal [5].

Acute kidney injury (AKI) is associated with inflammation. In patients with established AKI, serum interleukin (IL)-6, IL-8, IL-10, and tumor necrosis factor-α (TNF-α) were increased [6–9]. These cytokines have deleterious effects to kidneys and heart [10–13]. Elevated IL-8 levels have been associated with AKI in patients undergoing liver transplants, patients in septic shock, and patients with acute lung injury [14, 15]. It is believed that continuous renal replacement therapy (CRRT) can not only maintain the water balance and excrete the metabolic products but also mitigate the inflammation and promote kidney recovery. CRRT can remove the inflammatory cytokines to regulate the metabolic adaption in kidney and restore the kidney recovery to protect the kidney in septic AKI [16].

The anticoagulants are usually required during (CRRT) to maintain circuit patency. Systemic heparin remains the previously anticoagulant used for CRRT. However, heparin may pose an increased risk of bleeding especially in some groups of patients such as post-operative, liver failure and the development of heparin-induced thrombocytopenia [2, 17]. Regional citrate anticoagulation (RCA) is an attractive alternative in critically ill patients.

There are conflicting results regarding the effect of RCA on the cytokine levels [18–24]. No study has analyzed the cardiac effects of citrate CRRT. One animal study found that venovenous CRRT in 34 immature pigs decreased the intrathoracic blood volume, stroke volume index and central venous press use (CVP) but increased the systemic vascular resistance index and left ventricular contractility [25]. In critically ill patients, hypotension is usually observed, particularly at the beginning of CRRT. The initial hypotension is related to hypovolemia. However, metabolic acidosis contributes to persistent hypotension thereafter. Citrate can eliminate acidosis and thus help prevent hypotension [26]. This study aimed to evaluate the effect of citrate CRRT on cardiac function, such as cardiac output (CO), systemic vascular resistance (SVR), cardiac index (CI), systemic vascular resistance index (SVRI) and central venous pressure (CVP), compared with the heparin-free protocol. This study also explored the effect of RCA on improvements in inflammatory cytokines, filter life span, metabolic derangement and patient and renal survival at 28 days in both the RCA and heparin-free groups.

## Materials and methods

This study was a prospective, multicenter, open-label randomized trial conducted at two intensive care units (ICUs), King Chulalongkorn Memorial Hospital, Vajira Hospital, Bangkok, Thailand, from February 2019 to November 2020. The Institutional Ethics Boards of the participating centers approved the protocol. Written informed consent was obtained from either a close relative or a legal representative of each patient. Coinvestigators at each participating site were responsible for enrolling the patients and completing the case record form. The trial is registered at Clinicaltrial.gov as NCT 04,865,510.

### Study population

Adult critically ill patients with stage 3 AKI (defined by KDIGO 2012 criteria) were screened. The inclusion criteria were as follows: requirement for CRRT, age older than 18 years, and no contraindication to CRRT. We excluded patients with any of the following criteria: baseline serum creatinine > 2 mg/dL (male) or > 1.5 mg/dL (female), a history of renal transplantation, known pregnancy, a previous dialysis within 30 days, severe liver disease, end-stage heart disease or untreatable malignancy, a moribund status with expected survival less than 30 days, previous use of heparin or other anticoagulant, antiplatelet therapy within 7 days except for deep vein thrombosis, active bleeding at the time of enrollment and/or severe coagulopathy, receiving blood or blood components before enrollment, a hemoglobin level less than 7.5 g/dL and/or platelet count less than 100,000/mm^3^, previous underlying clotting disorders such as a hypercoagulable state, severe malnutrition (body mass index less than than 18), and CRRT for reasons other than acute AKI.

### Sample size calculation

A previous report by Chowdhury et al. [27] indicated that the filter half-life was 26 h in the RCA group (interquartile range (IQR) = 10–52) and 12 h in the non-RCA group (IQR = 6–28). The values in the formula were replaced by comparing two independent groups. The calculated sample size was 48 in each arm. As a pilot study, we recruited 20 patients in each arm to detect the difference in the cardiac effect between the RCA and heparin-free groups.

### Randomization

After screening for the eligible criteria for inclusion, all the included participants who agreed with this study were randomly assigned by systemic random sampling (block of four). Group A was the citrate group, and group B was the heparin-free group.

### Interventions

#### RCA group

CRRT was performed using Prismaflex (Baxter Healthcare/Gambro Spain) or an Infomed machine (HF440, Infomed SA, Switzerland) with a citrate pump. The functional mode was continuous venovenous hemodiafiltration (CVVHDF) in the postdilution mode with ST 150 filter sets. The dialysate was Accusol or Prism0cal B22. The dose of CRRT was 20–25 ml/kg/hr, with a blood flow of 150–200 ml/min. If the patient had hypercatabolic state such as severe metabolic acidosis, we would increase the dose of CRRT to 35–40 mL/kg/hr. If metabolic acidosis still persisted, we would adjust the bicarbonate in the infusate and/or replacement fluid to personalize the CRRT parameters. Trisodium citrate solution (4%; 136 mmol/L) was infused into the arterial line before the blood pump at a dose of 4 mmol/L of plasma flow. Calcium chloride (5% of 340 mmol/L elemental calcium) was infused into the venous return to maintain systemic ionized calcium in the normal range (1.0–1.20 mmol/L), and the target values for ionized calcium (iCa^2+^) after the dialysis membrane were 0.25–0.35 mmol/L. The rate of calcium infusion was adjusted in a timely manner based on repeated measurements of the calcium concentration. The detailed calcium supplementation protocol is shown in the additional file.

#### Heparin-free group

The circuit was periodically flushed with 50 ml of saline via the access limb every 30 min. When the prefilter pressure started to rise, additional saline flushes were given.

### All the protocol of intervention are in the supplemental file

#### Data collection

We followed all the patients from day 0 (start CRRT) for 28 days or until hospital discharge. We collected patient plasma at the start of the study (day 0) and on days 3, 5, 7, 14, and 28. The samples were used to measure inflammatory markers (Il 6, IL 8, IL 10 and TNFα). Plasma was collected using EDTA as an anticoagulant and then centrifuged for 10 min at 3,000 rpm. The samples were then stored in aliquots at ≤ − 80 °C. Repeated freeze–thaw cycles were avoided.

The Magnetic Luminex^®^ Performance Assays (R and D Systems, Minneapolis, USA) allows multianalyte analysis of biomarkers in a single complex sample using the Bio-Rad Bio-Plex System (Bio-Rad Laboratories, Hercules, USA). The output was used according to the manufacturer’s instructions. The following cytokines were measured: IL-1β, IL-6, IL-8, IL-10, and TNF α. The amounts of cytokines were calculated by comparison with the standard curve.

**Fig. 1.**
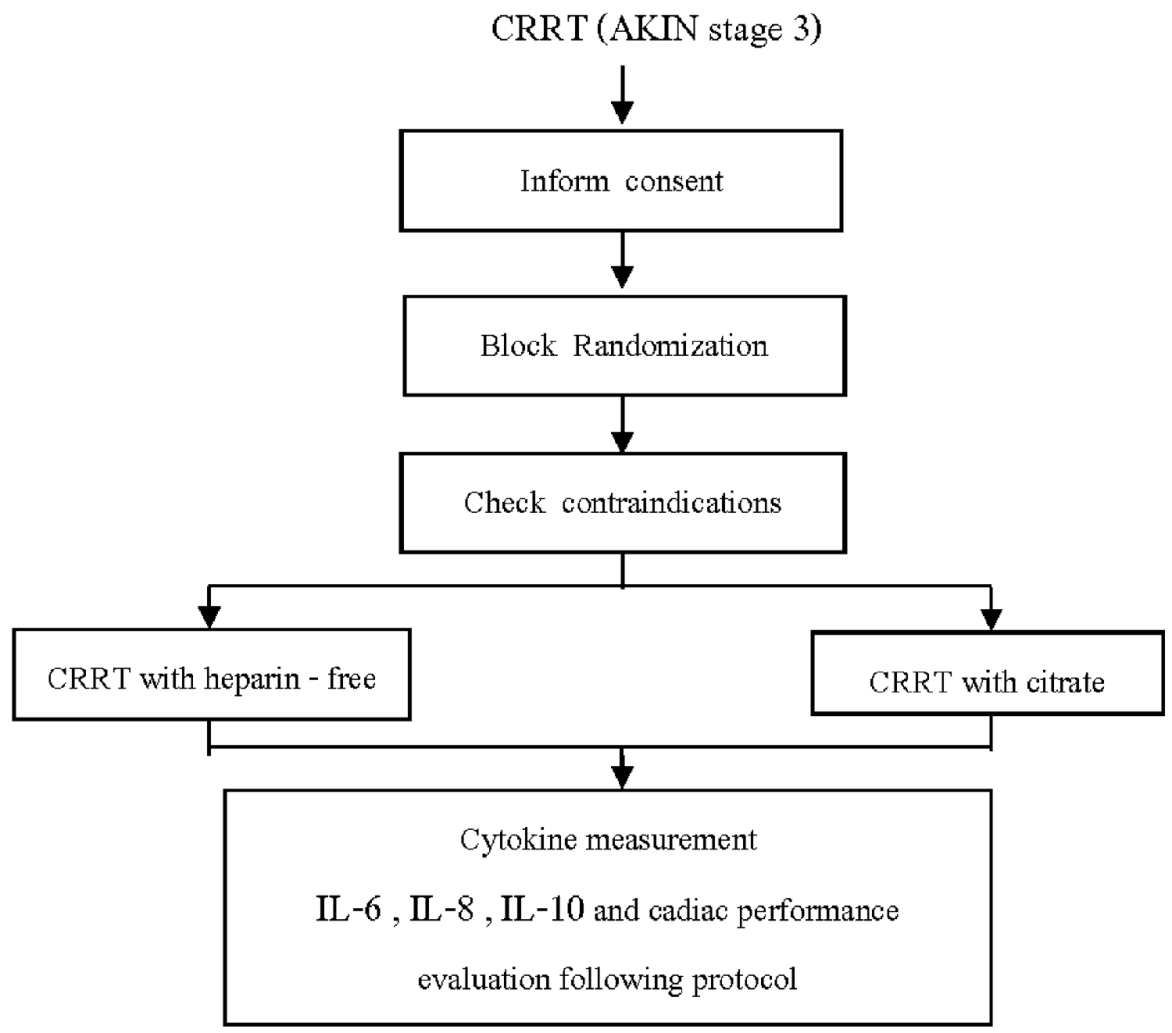
Study flowchart

The markers of dialysis efficiency (BUN, creatinine), other parameters related to AKI (acid base status, calcium, phosphorus, and hemoglobin), adverse events, dialysis clotting, the hemodynamic status, the duration of mechanical ventilation, and inotropic support were evaluated. The hemodynamic parameters were monitored using an EV 1000 clinical platform. After cannulating the arterial line at the appropriate site, the line was connected to the FloTrac Sensor/EV1000™ Clinical Platform (Edwards Lifescience, Irvine, CA, USA). This system provides a minimally invasive arterial waveform analysis to obtain cardiac performance data, which include the cardiac output (CO), cardiac index (CI), stroke volume (SV), and stroke volume indexes (SVI). Additionally, it provides information about systemic vascular resistance (SVR). Thus, the patient hemodynamic parameters as mentioned above were measured at the following 6 time points: after the initiation of CRRT (T1), every 6 h later for 24 h (T2, T3, T4, T5), and at hour 72 (T6).(Fig. 1) The dose of vasoactive/vasopressor agents is expressed as the catecholamine index, a dimensionless variable calculated as: (dopamine dose1)(dobutamine dose1) (adrenaline dose100) (noradrenaline dose100) (phenylephrine dose100), wherein all doses are expressed as µg/kg/min.The higher the score, the greater the vasopressor requirement [28].

#### Follow-up

Our protocol aimed to keep postfilter ionized calcium at 0.25–0.35 mmol/L. We monitored postfilter iCa, prefilter iCa, prefilter total calcium and acid–base balance at 1 min and then every 6 h. We also measured BUN, creatinine, phosphate, and magnesium daily.

### Statistical analysis

Data analysis was performed using SPSS version 22. All the data were subjected to the D’ Agostino-Pearson’s test to assess variable distribution, and continuous nonnormally distributed variables were analyzed using the Mann–Whitney test. Normally distributed variables were analyzed using two-tailed paired *t* test or one-way ANOVA and Bonferroni’s multiple comparison test. A *p* value < 0.05 was considered statistically significant. The filter life span was compared between two treatment arms in the intention-to-treat principle. The primary outcomes (CI and SVR) were analyzed as repeated measurement analysis. Overall survival for all patients was estimated by the Kaplan–Meier method. Categorical data were described as numbers and percentages. Continuous variables were described as means (with standard deviation, SD) or medians (with interquartile range, IQR).

## Results

Of the 41 recruited patients, 20 were from King Chulalongkorn Memorial Hospital and 21 patients were from Vajira Hospital. Their characteristics are presented in Table 1. Twenty-five (60.58%) were male, the mean age was 66.02 ± 15.62 years, and the mean body mass index was 23.97 ± 5.61. The Acute Physiology and Chronic Health Evaluation Score (APACHE) II of 23.73 ± 8.39 and was not different between the two groups. The main cause of AKI was sepsis (*n* = 29, 70.73%). The remaining causes were ischemia (*n* = 10), nephrotoxicity (*n* = 1, 2.43%), cardiorenal syndrome (*n* = 1, 2.43%) and multifactorial causes (*n* = 4, 9.75%).

**Table 1:** Study population characteristics at enrollment

	Total	Heparin-free (*n* = 20)	citrate (*n* = 21)	*p* value
	*n* (%) | Mean ± SD	*n* (%) | Mean ± SD	*n* (%) | Mean ± SD	
Study site				0.636
King chulalongkorn memorial hospital	20 (48.78)	9 (45.00)	11 (52.38)	
Vajira hospital	21 (51.22)	11 (55.00)	10 (47.62)	
Male gender	25 (60.98)	11 (55.00)	14 (66.67)	0.444
Mean age (Year)	66.02 ± 15.62	65.75 ± 17.24	66.29 ± 14.34	0.914
Underlying conditions				
Diabetes mellitus (%)	9 (21.95)	3 (15.00)	6 (28.57)	0.454
Hypertension (%)	23 (56.10)	9 (45.00)	14 (66.67)	0.162
Dyslipidemia (%)	19 (46.34)	7 (35.00)	12 (57.14)	0.155
Ischemic heart disease (%)	7 (17.07)	2 (10.00)	5 (23.81)	0.410
Peripheral arterial disease (%)	2 (4.88)	0 (0%)	2 (9.52)	0.488
Malignancy (%)	11 (26.83)	4 (20)	7 (33.33)	0.335
HIV infection (%)	2 (4.88)	1 (5.00)	1 (4.76)	1.000
Alcoholism (%)	5 (12.20)	1 (5.00)	4 (19.05)	0.343
Chronic liver disease (%)	5 (12.20)	3 (15.00)	2 (9.52)	0.663
Height (cm)	162.71 ± 8.28	162.75 ± 8.32	162.67 ± 8.45	0.975
Mean actual body weight (Kg)	63.44 ± 14.99	63.29 ± 14.68	63.59 ± 15.65	0.951
Mean ideal body weight (Kg)	58.39 ± 10.66	58.80 ± 9.92	58.00 ± 11.54	0.814
Mean body mass index (kg/m2)	23.97 ± 5.61	23.87 ± 5.18	24.06 ± 6.12	0.916
Diagnosis of sepsis at enrollment	29 (70.73)	15 (75.00)	14 (66.67)	0.558
Mean APACHE score at enrollment	23.73 ± 8.39	24.74 ± 8.55	22.81 ± 8.34	0.475
Mean SOFA score at enrollment	13.10 ± 3.45	13.11 ± 2.51	13.10 ± 4.18	0.993
Mechanical ventilation				
Invasive	39(95.12)	20 (100.00)	19 (90.48)	0.488
Non-invasive	2(4.87)	–	2 (9.52)	
Mean serum bicarbonate at enrollment (mEq/L)	16.54 ± 6.76	15.55 ± 7.30	17.48 ± 6.23	0.368

Twenty patients were randomized into the heparin-free group, and 11 patients were randomized into the citrate group. The randomization was well balanced between the treatment groups. All the patients had stage 3 AKI and fulfilled the criteria for CRRT therapy.

### Cardiovascular parameters

The cardiac performance data from the EV1000 are provided in Table 2. The cardiac output (CO), cardiac index (CI), systemic vascular resistance (SVR) and systemic vascular resistance index (SVI) were not significantly different between the heparin-free and citrate groups at any time point. The parameters were also not different from baseline values. The mean arterial blood pressure did not significantly differ between the groups. However, during CRRT, the MAP in the citrate group was higher than that in the heparin-free group (81.70 ± 10.88 mmHg vs 75.27 ± 11.80; *p* = 0.927) mmHg 18 h after commencing CRRT. The central venous pressure decreased in both groups as ultrafiltration was performed to remove excessive fluid (16.83 ± 7.87 cmH_2_O to 11.75 ± 11.69 cmH_2_O in the group at 72 h.). We also calculate the cathecholamine index (CAI) and did not find any significant different of CAI in both groups.

**Table 2:** Hemodynamic parameters measured by EV-1000 FLO-TRAC

	*n*	Baseline	6 h	12 h	18 h	24 h	72 h	*p* value*	*p* value**
		Mean ± SD	Mean ± SD	Mean ± SD	Mean ± SD	Mean ± SD	Mean ± SD		
CI									
Heparin-free	18	7.34 ± 15.62	5.15 ± 7.69	4.65 ± 6.01	4.70 ± 6.33	4.66 ± 5.20	4.85 ± 5.25	0.295	0.214
Citrate	20	3.16 ± 1.41	2.99 ± 1.23	3.07 ± 1.36	3.06 ± 1.03	3.01 ± 1.30	2.99 ± 1.03	0.894	
CO									
Heparin-free	20	5.52 ± 3.11	5.14 ± 2.64	5.01 ± 2.45	4.77 ± 2.47	5.21 ± 2.72	5.23 ± 2.67	0.243	0.759
Citrate	21	5.11 ± 2.16	5.05 ± 2.36	4.78 ± 1.97	4.84 ± 1.75	4.80 ± 2.23	5.00 ± 2.20	0.734	
SVR									
Heparin-free	20	1072.45 ± 497.69	1273.35 ± 824.83	1188.90 ± 610.72	1183.10 ± 698.12	1212.50 ± 713.20	1270.80 ± 824.55	0.535	0.133
Citrate	20	971.80 ± 490.14	935.75 ± 516.50	954.85 ± 487.33	944.10 ± 448.94	935.00 ± 453.66	923.10 ± 443.62	0.865	
SVI									
Heparin-free	19	37.00 ± 22.50	33.26 ± 18.58	30.95 ± 14.73	30.84 ± 15.84	33.63 ± 16.95	33.21 ± 17.58	0.203	0.796
Citrate	21	31.86 ± 18.03	31.81 ± 16.58	32.38 ± 16.51	31.09 ± 14.30	31.19 ± 16.19	32.71 ± 17.26	0.922	
CVP									
Heparin-free	19	15.95 ± 6.94	17.32 ± 7.36	16.32 ± 5.84	16.16 ± 7.68	16.32 ± 6.96	15.68 ± 6.79	0.588	0.861
Citrate	19	16.37 ± 7.37	17.95 ± 6.96	17.05 ± 7.63	16.16 ± 6.51	16.26 ± 7.30	16.16 ± 7.48	0.485	
MAP									
Heparin-free	20	72.00 ± 11.23	74.82 ± 13.55	77.18 ± 14.96	75.27 ± 11.80	77.55 ± 13.95	73.91 ± 15.21	0.792	0.927
Citrate	21	72.30 ± 17.43	71.30 ± 10.72	78.90 ± 11.83	81.10 ± 10.88	73.37 ± 13.69	75.70 ± 15.28	0.435	
CAI									
Heparin-free	20	30 (14.5–51.5)	9.5 (1–38)	0 (0–10)	0 (0)	0 (0)	0 (0)		0.35
Citrate	20	51.7 (33.75–99.5)	35 (0–76.7)	7.5 (0–23)	0 (0–19)	0 (0	0 (0)		

^*^One-way repeated measures ANOVA

^**^Two-way repeated measures ANOVA

Significant if *p* < 0.05

*CI* cardiac index, *CO* cardiac output, *SVR* systemic vascular resistance, *SVI* systemic vascular resistance index, *CVP* central venous pressure, *MAP* mean arterial pressure, *CAI* catecholamine index

### Acid–base balance

Overall, both treatment arms successfully corrected metabolic acidosis compared with the baseline values. The baseline bicarbonate level was comparable in both groups (15.55 ± 7.30 mmol/L vs 17.48 ± 6.23 mmol/L, *p* = 0.368 in the heparin-free and citrate groups, respectively). By the end of 72 h, both groups had nearly normal bicarbonate levels (23.40 ± 3.05 mmol/L and 23.75 ± 1.26 mmol/L, pH 7.42 ± 0.05 and 7.44 ± 0.05 in the heparin-free and citrate groups, respectively; *p* = 0.032). Slight metabolic alkalosis developed more frequently in the citrate group at a certain time point (24 h.) (28.00 ± 2.58 mmol/L). The bicarbonate levels returned to normal values more in the heparin-free group (∆HCO_3_ 10.60 ± 5.41 VS 4.43 ± 2.57 *p* = 0.024 in the heparin-free and citrate groups, respectively).

### Cytokine levels

The serum Il-1β, Il-6, Il-8, Il-10 and TNF-α were measured at baseline and 72 h after CRRT. The baseline levels of plasma Il-6 and Il-8 were significantly lower in the heparin-free group than in the citrate group (Table 3). At 72 h, these biomarkers decreased from baseline but were not statistically significant. No significant differences were found in these biomarkers on day 3 within and between treatment arms.

**Table 3:** Mean change of Inflammatory cytokine levels

	Total (*n* = 41)	Heparin-free (*n* = 20)	citrate (*n* = 21)	*p* value
	Mean ± SD	Mean ± SD	Mean ± SD	
Day 1 (ng/mL)				
IL-1B	59.59 ± 243.99	9.17 ± 18.22	107.60 ± 337.33	0.197
IL-6	6477.16 ± 13,081.35	1227.39 ± 1946.31	11,476.95 ± 16,877.11	0.012
IL-8	2940.34 ± 6528.43	691.17 ± 790.51	5082.41 ± 8646.99	0.031
IL-10	854.02 ± 2514.74	149.14 ± 221.9	1525.33 ± 3410.43	0.080
TNF-alpha	57.45 ± 104.31	29.80 ± 18.79	83.77 ± 141.19	0.097
Day 3 (ng/mL)				
IL-1B	48.32 ± 240.99	4.66 ± 5.16	89.90 ± 335.27	0.263
IL-6	5287.01 ± 12,634.76	843.95 ± 1166.46	9518.49 ± 16,716.46	0.028
IL-8	2660.97 ± 6482.16	634.02 ± 803.22	4591.4 ± 8683.460	0.050
IL-10	667.36 ± 2462.73	107.73 ± 125.45	1200.34 ± 3391.71	0.156
TNF-alpha	47.50 ± 100.81	29.00 ± 20.76	65.12 ± 138.73	0.252

Independent *t* test

Significant if *p* < 0.05

### Filter life span

The mean circuit lifetime was comparable in both groups, although the maximum lifetime (the maximum duration of time for the circuit that can run without clotting) in the circuit groups was longer in citrate group (44.64 ± 26.56 h. vs 36.63 ± 23.48 h in the citrate and heparin-free groups, respectively; *p* = 0.421). The heparin-free group changed filters slightly more frequently than the citrate group (1.55 ± 0.76 vs 1.37 ± 0.60 filters in the heparin-free and citrate groups, respectively; *p* = 0.42; Table 4).The postfilter ionized calcium was within the therapeutic range at every time point (data in the supplemental file).

**Table 4:** Circuit lifetime (hr)

	Total (*n* = 39)	Heparin-free (*n* = 20)	citrate (*n* = 19)	*p* value
Mean				
Mean ± SD	40.53 ± 25.04	36.63 ± 23.48	44.64 ± 26.59	
Median (IQR)	35 (18–68)	30.5 (17.25–57)	48 (18–72)	0.421
Minimum				
Mean ± SD	36.74 ± 26.56	31.05 ± 25.05	42.74 ± 27.45	
Median (IQR)	24 (13–68)	22.5 (10.5–53)	48 (16–72)	0.214
Maximum				
Mean ± SD	44.64 ± 26.53	42.7 ± 26.55	46.68 ± 27.08	
Median (IQR)	48 (22–72)	36 (18.5–72)	58 (22–72)	0.842
Count				
Mean ± SD	1.46 ± 0.68	1.55 ± 0.76	1.37 ± 0.60	
Median (IQR)	1 (1–2)	1 (1–2)	1 (1–2)	0.421

Mann–Whitney *U* test

Significant if *p* < 0.05

### Safety

Four cases (9.75%) showed citrate accumulation (high anion gap metabolic acidosis and low plasma calcium), and one case (2.4%) had pre-existing liver dysfunction. For these four patients, therapy decreased the citrate flow rate in one case, and the clinical situation eventually improved. One patient had underlying liver disease with high transaminase levels, and citrate administration was terminated. CRRT was continued without heparin for 6 h before the patient died. The total calcium to ionized calcium (T/iCa^4^) was more than 2.5 in these cases.

The most frequent complications were hypernatremia (14%), hypocalcemia (7%), and hypomagnesemia (36.57%). No clinically significant hemorrhage was observed in the RCA group. Severe hypocalcemia (iCa^2+^ < 0.9 mmol/L) was not observed. We found one case in the heparin-free group with upper gastrointestinal bleeding that was resolved with medical therapy.

### Mortality

**Fig. 2:**
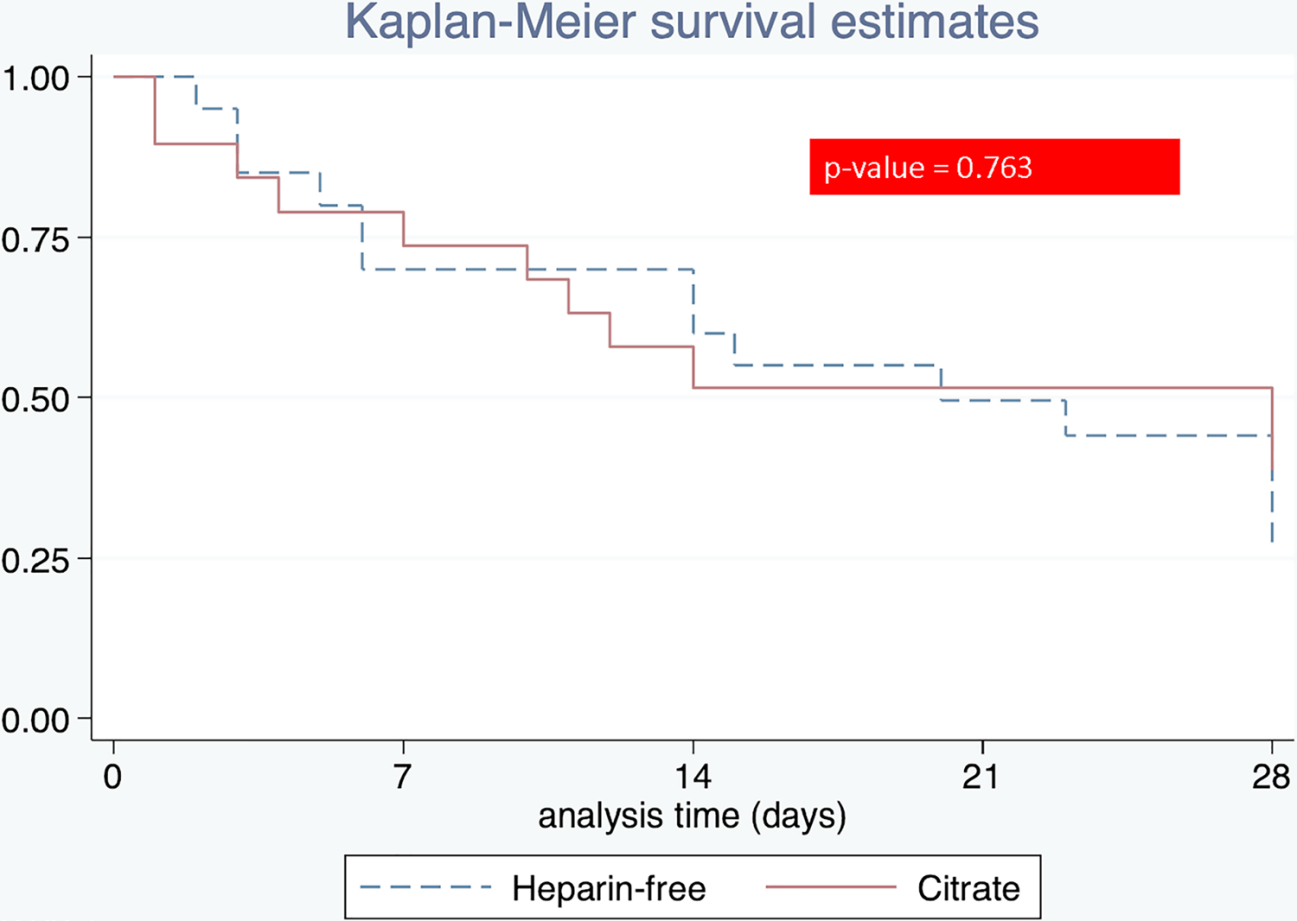
Survival curves of CRRT patients receiving heparin-free and citrate anticoagulants (straight line, citrate group; dashed line, heparin-free group). The figure shows the Kaplan–Meier curve of the probability of survival from randomization to day 28. *CI* confidence interval, *HR* hazard ratio

Among the 41 patients included in the study, 25 (60.97%) died while in the ICU (overall in-hospital mortality, 65.85%; Table 5). The median ICU length of stay was 8 days (3–24 days), and the median hospital length of stay was 14 days (5–40 days). Among survivors, 13 (32.50%) required RRT at 28 days after commencing dialysis. No significant differences were found in dialysis dependence on day 28 and renal recovery between the treatment arms (Fig. 2).

**Table 5:** Clinical outcomes

Mortality, survival	Citrate median (IQR)	Heparin-free Median (IQR)	*p* value
ICU mortality, *n* (%)	12 (57.14)	13 (65.00)	0.606
ICU length of stay (days), median (IQR)	11 (3–27)	6 (3–23)	0.480
Hospital length of stay (days), median (IQR)	12 (35–38)	15 (6–40)	0.465
28 day mortality *n* (%)	13 (61.90)	14 (70.00)	0.585
28 day dialysis status *n* (%)	8 (38.10)	5 (26.32)	0.427

### Lactate level

**Fig. 3:**
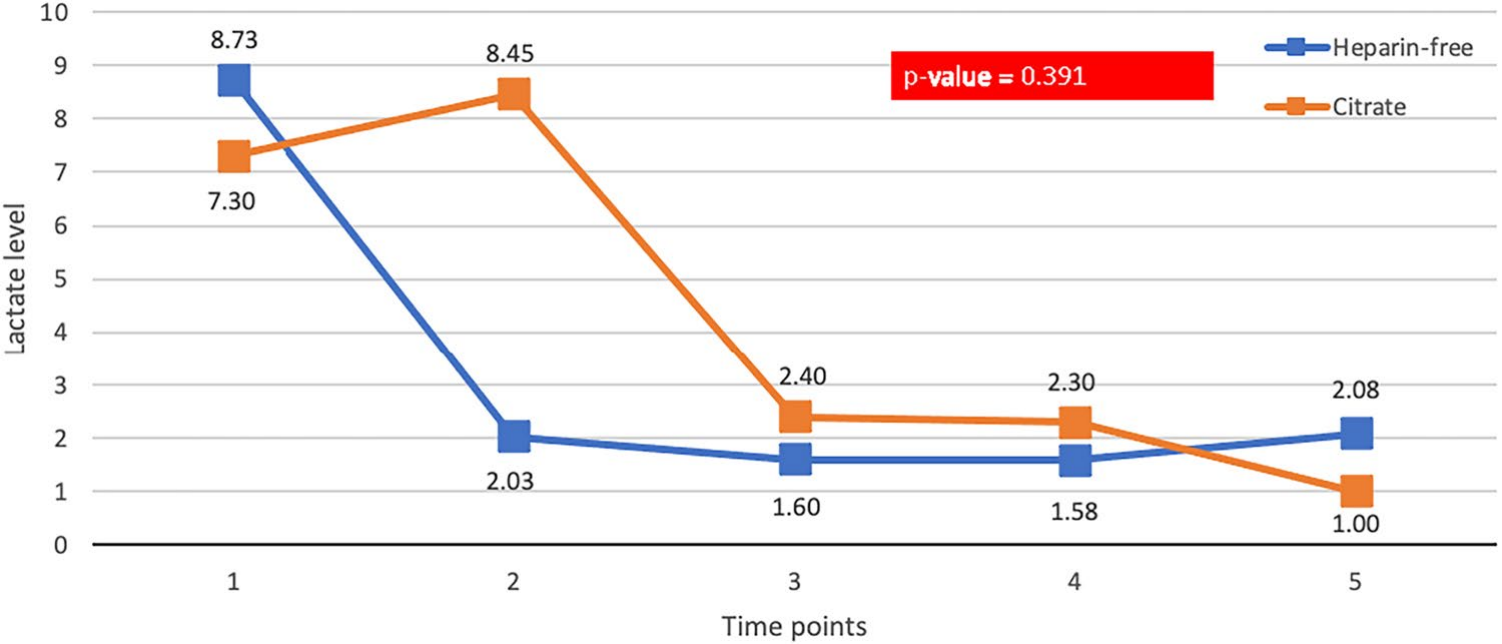
Lactate level of CRRT patients receiving heparin-free and citrate anticoagulants

After commencing CRRT, the blood lactate level decreased nonsignificantly from the baseline values in both groups. The mean differences in lactate from t (time) 72 h compared with baseline were − 6.10 ± 5.85 mmol/L vs − 4.23 ± 5.44 mmol/L in the heparin-free vs citrate group (*p* = 0.631; Fig. 3).

### Hemodynamic and respiratory support

**Fig. 4:**
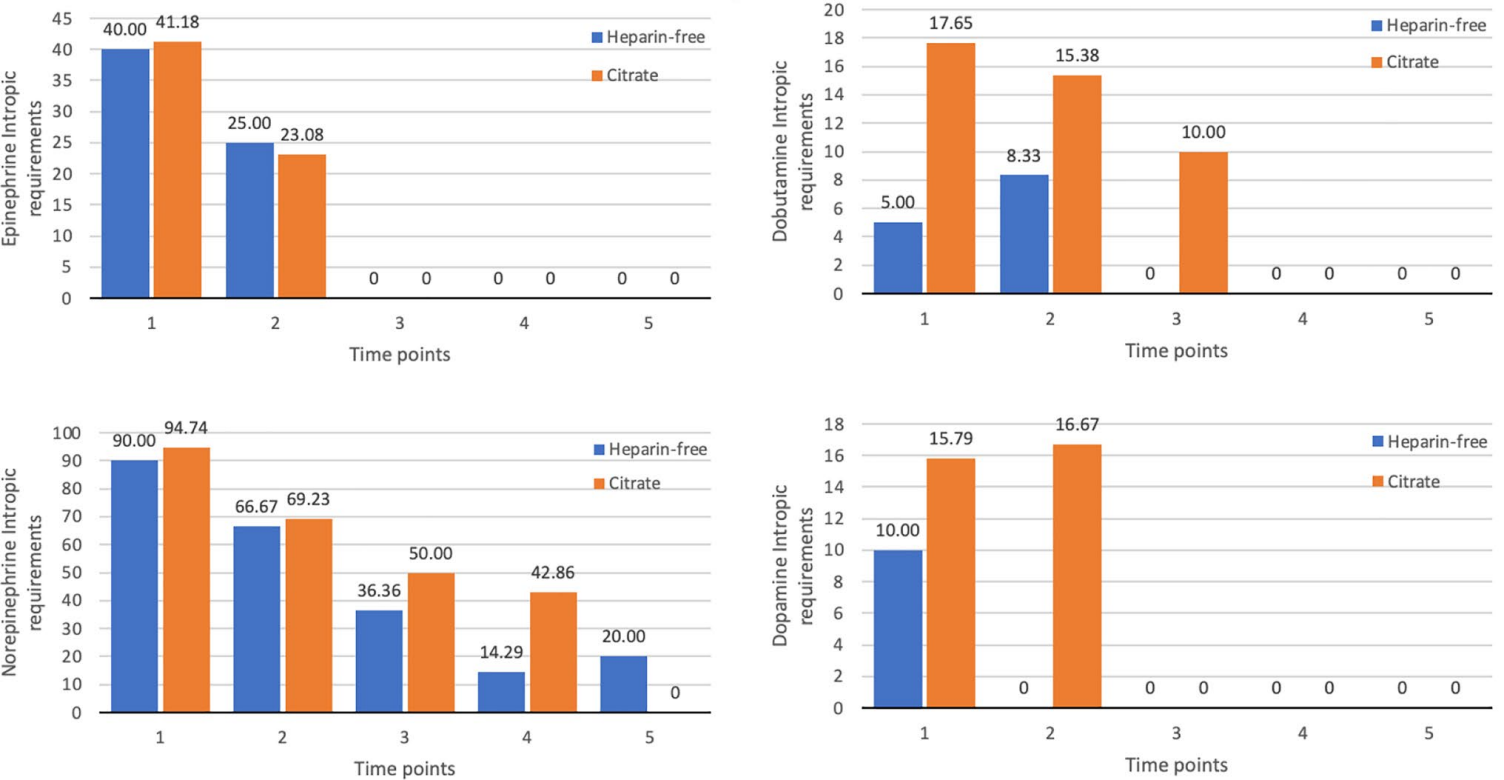
Hemodynamic parameters of CRRT patients receiving heparin-free and citrate anticoagulants

More than 50% of all patients required inotropic support at baseline (Fig. 4). Most received norepinephrine (*n* = 36, 92.31%). The remaining inotropic drugs were epinephrine (*n* = 15, 40.54%), dobutamine (*n* = 4, 10.81%) and dopamine (*n* = 5, 72.82%). The proportion of patients who required inotropic support was not different between the citrate and heparin-free groups. The respiratory support requirements were also comparable between the two treatment arms. Only two cases used non-invasive respiratory support in the citrate group. (Fig. 4).

## Discussion

Citrate anticoagulates the extracorporeal circuit by chelating ionized calcium. Currently, the KDIGO recommends citrate as the first-line anticoagulation agent over heparin for CRRT in patients without citrate contraindication [5]. From our study, the baseline characteristics such as the cardiac performances, the level of inflammatory cytokines were not significantly different between the groups at any time point. Although CRRT can affect hemodynamic impact mainly form decreased intravascular volume and underlying cardiac problems, our patients group has stable hemodynamic parameters in both groups at every time point. The citrate group had tendency to have longer filter life time than heparin-free group although not statistically significant. The maximum filter survival time was insignificantly longer in the RCA group than in the heparin-free group. No serious side effects were observed for either treatment arm, even in the group of liver dysfunction patients.

A previous report showed that citrate stabilized hemodynamics during CRRT and could have renal protection during early postischemic AKI [26]. Citrate also is superior in terms of safety and efficacy, with longer filter life span than heparin for continuous renal replacement therapy in critically ill children [29].However, citrate causes a decrease in arterial blood pressure [30]. Additionally, together with the hemodynamic response to CRRT (diminished CO, secondary to SVRI increase, and decreased cardiac filling pressure) [25], citrate can, in contrast, be harmful to the patient. We demonstrated that citrate anticoagulation did not have adverse effects on hemodynamics, cardiac output, or the cardiac index systemic vascular resistance index. Our findings confirmed the safe use of citrate in critically ill patients even if some patients had liver impairment (*n* = 5, 12.20%). We chose the heparin-free protocol since this is the usual practice in our units and lessen the bleeding risks that can be encountered in the critically ill patients. Furthermore, the difference in heparin dosages among each patient may confound the filter lifespan interpretation in the trial.

Citrate positively affects mortality and renal recovery [26], possibly because of interference with inflammation and oxidative stress. The levels of cytokines (IL-6, IL-8, IL-10, IL-1β, TNFα), either in serum or urine, are elevated in AKI. Higher levels of these cytokines are associated with longer hospital stays and ventilator length [31]. Conflicting results exist on the circulating levels of these cytokines in CRRT patients who received RCA. Gattas et al. [19] found similar effects on the serum IL-6, IL-8 and IL-10 levels between citrate and heparin anticoagulation. Tirathanagul et al. [20] reported a significant decrease in systemic prefilter myeloperoxidase (MPO) and IL-8 in the CVVH with citrate group, whereas heparin provided only a significant TNF-α reduction; however, no survival benefit was identified. Citrate anion modulates oxidative metabolism, systemic inflammation and vascular function, increases glutathione production, and reduces complement and neutrophil activation [21]. We have demonstrated that CRRT effectively reduces these cytokine levels significantly from baseline, as explained by cytokine removal using the convective modalities of dialysis. However, citrate showed no benefit over the heparin-free group. The comparable dose delivery in both treatment arms might explain the similar clearance of the inflammatory cytokine in both modalities (Table 5).

Almost all the studies reported a significantly longer filter life span in the RCA group [32, 33]. Our studies demonstrated prolonged filter survival in the citrate group but insufficient power to show statistical significance. The reason could be the low number of cases in this study. More than 50% of our cases performed using the postdilution mode support the notion that postfilter replacement does not offset the advantageous effect of RCA on filter survival (Table 4). There was no difference in the rate of catheter malfunction between both groups. The actual delivered effluent was comparable in both groups.

Clotting was the main cause of circuit change in both the heparin-free and RCA groups. However, the scheduled filter change was more common and clinically relevant with the heparin-free group than with the citrate group (1.55 ± 0.76 vs 1.37 + 0.60 filters in the heparin-free and citrate groups, respectively; *p* = 0.421), although the difference was not statistically significant. The incidence of bleeding was comparable in both groups, although one case in the heparin-free group had severe GI bleeding.

The most common metabolic complication related to citrate is metabolic alkalosis because of citrate metabolism to bicarbonate in the liver. In our study, metabolic complications were mild in all patients. Only hypernatremia was observed in only two cases and was not related to liver function impairment. The citrate flow rate might be too high (150 ml/h) in this case. Five patients with chronic liver diseases tolerated RCA very well. This finding is consistent with that in a study from Klingele et al. [34] who demonstrated that citrate can be used in most cases with impaired liver function. They hypothesized that adequate citrate metabolism must be possible outside the liver. Taken together, hepatic dysfunction does not seem to be an obstacle to using citrate for CRRT. To date, a reduction in mortality has not been demonstrated in most studies [35]. We did not encounter any differences in mortality between the two arms, likely because of the low number of participants, resulting in underpower in the statistical analysis. Recently, the large clinical trial, the RICH Trial which included up to 1450 critically ill patients with AKI requiring CRRT is currently underway to investigate the effect of RCA on filter life span and overall survival in a 90 day as compared with systemic heparin anticoagulation but did not examine hemodynamics as primary end point [36].

We acknowledge some limitations of our study. First, various CRRT machines were used, and some designs had heterogeneity in the pre- and postdilution mode (Infomed machine can only use the postdilution mode) that might occur after the circuit lifetime. However, the CRRT doses were equivalent, possibly reducing the clinical heterogeneity. Second, the number of participants was small (20 in each arm), and more than 50% died within 7 days. Third, there were diverse patient groups: leading to more missing data and an insufficient power for the outcome and diverse levels of inflammatory cytokines. However, our study demonstrated the consistent reduction of these cytokines after CRRT in both arms, indicating the powerful effect of CRRT in reducing inflammation, more than the effect of citrate per se. Fourth, the minimally invasive hemodynamic measurement become less accurate with changes in vascular tone and reactivity (for example, during pharmacologically induced vasoconstriction) [37]. Finally, several intrinsic patient characteristics (valvular disease, arrhythmias) and more importantly, hospital course treatments (ventilation, vasopressors, inotropes, etc.) can impact hemodynamics. More sample sizes is needed to overcome the evenly distribute influential variables across the two arms and report on the impact of citrate on hemodynamics.

## Conclusions

We found that RCA did not affect hemodynamic or cardiovascular changes during CRRT and produced a longer circuit survival time, although not statistically significant. The CO, CI, and SVRI stabilized during the treatment period, while fluid was continually removed (confirmed by CVP reduction). The adverse effects of citrate were minimal even in patients with liver dysfunction. CRRT effectively reduced inflammatory cytokines and may be beneficial in restoring renal function. The 28 day mortality was comparable. RCA is a valid alternative to traditional anticoagulation that is heparin free, because it reduces the bleeding risk and maintains the hemodynamic status with minimal adverse effects.

## Supplementary Information

Below is the link to the electronic supplementary material.

Supplementary file1 (DOC 43 KB)

## Data Availability

All the data generated and/or analyzed during this study are included in the published article.

## Abbreviations

AKI: Acute kidney injury
APACHE II: Acute Physiology and Chronic Health Evaluation
ACD: Acid citrate dextrose
CCC: Citrate calcium complex
CRRT: Continuous renal replacement therapy
CVP: Central venous pressure
CO: Cardiac output
CI: Cardiac index
iCa: Ionized calcium,
RCA: Regional citrate anticoagulation
SVR: Systemic vascular resistance
SVRI: Systemic vascular resistance index
TMP: Transmembrane pressure

## References

[1] Hoste EA, Bagshaw SM, Bellomo R, Cely CM, Colman R, Cruz DN (2015). Epidemiology of acute kidney injury in critically ill patients: the multinational AKI-EPI study. Intensive Care Med.

[2] Gatward JJ, Gibbon GJ, Wrathall G, Padkin A (2008). Renal replacement therapy for acute renal failure: a survey of practice in adult intensive care units in the United Kingdom. Anaesthesia.

[3] Kellum JA, Mehta RL, Angus DC, Palevsky P, Ronco C, ADQI Workgroup (2002). The first international consensus conference on continuous renal replacement therapy. Kidney Int.

[4] Uchino S, Bellomo R, Morimatsu H, Morgera S, Schetz M, Tan I, Bouman C, Macedo E, Gibney N, Tolwani A, Oudemans-van Straaten H, Ronco C, Kellum JA (2007). Continuous renal replacement therapy: a worldwide practice survey. The beginning and ending supportive therapy for the kidney (B.E.S.T. kidney) investigators. Intensive Care.

[5] Kidney Diseases (2012). Improving global outcomes (KDIGO) acute kidney injury work group. KDIGO clinicalpractice guideline for acute kidney injury. Kidney Int Suppl.

[6] Schrezenmeier EV, Barasch J, Budde K, Westhoff T, Schmidt-Ott KM (2017). Biomarkers in acute kidney injury - pathophysiological basis and clinical performance. Acta Physiol (Oxf).

[7] Kwon O, Molitoris BA, Pescovitz M, Kelly KJ (2003). Urinary actin, interleukin-6, and interleukin-8 may predict sustained ARF after ischemic injury in renal allografts. Am J Kidney Dis.

[8] Liangos O, Kolyada A, Tighiouart H, Perianayagam MC, Wald R, Jaber BL (2009). Interleukin-8 and acute kidney injury following cardiopulmonary bypass: a prospective cohort study. Nephron Clin Pract.

[9] de Fontnouvelle CA, Greenberg JH, Thiessen-Philbrook HR, Zappitelli M, Roth J, Kerr KF (2017). Interleukin-8 and tumor necrosis factor predict acute kidney injury after pediatric cardiac surgery. Ann Thorac Surg.

[10] Seghaye MC, Duchateau J, Grabitz RG, Faymonville ML, Messmer BJ, Buro-Rathsmann K, von Bernuth G (1993). Complement activation during cardiopulmonary bypass in infants and children. Relation to postoperative multiple system organ failure. J Thorac Cardiovasc Surg.

[11] Ataie-Kachoie P, Pourgholami MH, Morris DL (2013). Inhibition of the IL-6 signaling pathway: a strategy to combat chronic inflammatory diseases and cancer. Cytokine Growth Factor Rev.

[12] Ataie-Kachoie P, Pourgholami MH, Richardson DR, Morris DL (2014). Gene of the month: interleukin 6 (IL-6). J Clin Pathol.

[13] Kavsak PA, Ko DT, Newman AM, Palomaki GE, Lustig V, MacRae AR, Jaffe AS (2007). Risk stratification for heart failure and death in an acute coronary syndrome population using inflammatory cytokines and N-terminal pro-brain natriuretic peptide. Clin Chem.

[14] Liu D (2014). Using inflammatory and oxidative biomarkers in urine to predict early acute kidney injury in patients undergoing liver transplantation. Biomarkers.

[15] Sirota JC (2013). Urine IL-18, NGAL, IL-8 and serum IL-8 are biomarkers of acute kidney injury following liver transplantation. BMC Nephrol.

[16] Zhang J, Tian J, Sun H, Digvijay K, Neri M, Bhargava V, Yin Y, Ronco C (2018). How does continuous renal replacement therapy affect septic acute kidney injury?. Blood Purif.

[17] Oudemans-van Straaten HM, Kellum JA, Bellomo R (2011). Clinical review: anticoagulation for continuous renal replacement therapy–heparin or citrate?. Crit Care.

[18] Liu D, Huang P, Li X, Ge M, Luo G, Hei Z (2014). Using inflammatory and oxidative biomarkers in urine to predict early acute kidney injury in patients undergoing liver transplantation. Biomarkers.

[19] Gattas DJ, Rajbhandari D, Bradford C, Buhr H, Lo S, Bellomo R (2015). A randomized controlled trial of regional citrate versus regional heparin anticoagulation for continuous renal replacement therapy in critically ill adults. Crit Care Med.

[20] Tiranathanagul K, Jearnsujitwimol O, Susantitaphong P, Kijkriengkraikul N, Leelahavanichkul A, Srisawat N (2011). Regional citrate anticoagulation reduces polymorphonuclear cell degranulation in critically ill patients treated with continuous venovenous hemofiltration. Ther Apher Dial.

[21] Dellepiane S, Medica D, Guarena C, Musso T, Quercia AD, Leonardi G (2019). Citrate anion improves chronic dialysis efficacy, reduces systemic inflammation and prevents chemerin-mediated microvascular injury. Sci Rep.

[22] Pizzarelli F, Cantaluppi V, Panichi V, Toccafondi A, Ferro G, Farruggio S, Grossini E, Dattolo PC, Miniello V, Migliori M, Grimaldi C, Casani A, Borzumati M, Cusinato S, Capitanini A, Quercia A, Filiberti O, Dani L, Hephaestus study group (2021). Citrate high volume on-line hemodiafiltration modulates serum Interleukin-6 and Klotho levels: the multicenter randomized controlled study "Hephaestus". J Nephrol.

[23] Bellomo R, Tipping P, Boyce N (1993). Continuous veno-venous hemofiltration with dialysis removes cytokines from the circulation of septic patients. Crit Care Med.

[24] Hoffmann JN, Faist E (2001). Removal of mediators by continuous hemofiltration in septic patients. World J Surg.

[25] López-Herce J, Rupérez M, Sánchez C, García C, García E (2006). Effects of initiation of continuous renal replacement therapy on hemodynamics in a pediatric animal model. Ren Fail.

[26] Bienholz A, Reis J, Sanli P, de Groot H, Petrat F, Guberina H (2017). Citrate shows protective effects on cardiovascular and renal function in ischemia-induced acute kidney injury. BMC Nephrol.

[27] Chowdhury S, Lawton T, Akram A, Collin R, Beck J (2017). Citrate versus non-citrate anticoagulation in continuous renal replacement therapy: results following a change in local critical care protocol. I Intensive Care So.

[28] Tsujimoto H, Ono S, Hiraki S (2004). Hemoperfusion with polymyxin B-immobilized fibers reduced the number of CD16CD14 monocytes in patients with septic shock. J Endotoxin Res.

[29] Sık G, Demirbuga A, Annayev A, Citak A (2020). Regional citrate versus systemic heparin anticoagulation for continuous renal replacement therapy in critically ill children. Int J Artif Organs.

[30] Toyoshima S, Fukuda T, Masumi S, Nakashima Y, Kawaguchi Y, Nakayama M (2006). Maximum acceptable infusion rate of citrate: relationship between blood ionized calcium levels and cardiovascular effects in anesthetized rats. Clin Nutr.

[31] Liu KD, Altmann C, Smits G, Krawczeski CD, Edelstein CL, Devarajan P (2009). Serum interleukin-6 and interleukin-8 are early biomarkers of acute kidney injury and predict prolonged mechanical ventilation in children undergoing cardiac surgery: a case-control study. Crit Care.

[32] Grundström G, Christensson A, Alquist M, Nilsson LG, Segelmark M (2013). Replacement of acetate with citrate in dialysis fluid: a randomized clinical trial of short term safety and fluid biocompatibility. BMC Nephrol.

[33] Panichi V, Fiaccadori E, Rosati A, Fanelli R, Bernabini G, Scatena A, Pizzarelli F (2013). Post-dilution on line haemodiafiltration with citrate dialysate: first clinical experience in chronic dialysis patients. ScientificWorld Journal.

[34] Klingele M, Stadler T, Fliser D, Speer T, Groesdonk HV, Raddatz A (2017). Long-term continuous renal replacement therapy and anticoagulation with citrate in critically ill patients with severe liver dysfunction. Crit Care.

[35] Kindgen-Milles D, Brandenburger T, Dimski T (2018). Regional citrate anticoagulation for continuous renal replacement therapy. Curr Opin Crit Care.

[36] Meersch M, Küllmar M, Wempe C, Kindgen-Milles D, Kluge S, Slowinski T, Marx G, Gerss J, Zarbock A, SepNet Critical Care Trials Group (2019). Regional citrate versus systemic heparin anticoagulation for continuous renal replacement therapy in critically ill patients with acute kidney injury (RICH) trial: study protocol for a multicentre, randomised controlled trial. BMJ Open.

[37] Camporota L, Beale R (2010). Pitfalls in haemodynamic monitoring based on the arterial pressure. Crit Care.

