# Supplementary material for: The effect of citrate in cardiovascular system and clot circuit in critically ill patients requiring continuous renal replacement therapy
Source: J Artif Organs. 2022 Apr 12;26(1):53–64. doi: 10.1007/s10047-022-01329-0 (PMC9968675; doi:10.1007/s10047-022-01329-0)
Supplement: Supplementary file 1 — Supplementary file1 (DOC 43 KB) [file 10047_2022_1329_MOESM1_ESM.doc]

| **pH** |  |  |  |
| --- | --- | --- | --- |
|  | n | Mean ± SD | Median (IQR) |
| **Heparin-free** |  |  |  |
| time 1 | 20 | 7.33 ± 0.17 | 7.38 (7.31 - 7.42) |
| time 2 | 12 | 7.43 ± 0.09 | 7.45 (7.40 - 7.50) |
| time 3 | 10 | 7.39 ± 0.17 | 7.43 (7.35 - 7.50) |
| time 4 | 6 | 7.40 ± 0.21 | 7.46 (7.35 - 7.51) |
| time 5 | 5 | 7.42 ± 0.05 | 7.42 (7.37 - 7.46) |
| **Citrate** |  |  |  |
| time 1 | 21 | 7.36 ± 0.10 | 7.37 (7.29 - 7.44) |
| time 2 | 14 | 7.41 ± 0.12 | 7.43 (7.36 - 7.51) |
| time 3 | 8 | 7.46 ± 0.04 | 7.46 (7.42 - 7.50) |
| time 4 | 4 | 7.44 ± 0.05 | 7.43 (7.40 - 7.48) |
| time 5 | 3 | 7.44 ± 0.05 | 7.46 (7.38 - 7.47) |

**Table 1**: pH Value during the study period

| **post_filter iCa2+** |  |  |  |
| --- | --- | --- | --- |
|  | n | Mean ± SD | Median (IQR) |
| **Heparin-free** |  |  |  |
| time 1 | 1 | 1 | 1 |
| time 2 | 1 | 1 | 1 |
| time 3 | 0 | - | |
| time 4 | 0 | - | |
| time 5 | 0 | - | |
| **Citrate** |  |  |  |
| time 1 | 15 | 0.54 ± 0.16 | 0.54 (0.47 - 0.58) |
| time 2 | 12 | 0.55 ± 0.21 | 0.47 (0.42 - 0.56) |
| time 3 | 7 | 0.41 ± 0.06 | 0.41 (0.36 - 0.48) |
| time 4 | 7 | 0.40 ± 0.06 | 0.38 (0.35 - 0.43) |
| time 5 | 3 | 0.42 ± 0.03 | 0.44 (0.38 - 0.44) |

**Table 2**: Post-filter ionized calcium at each time point during the study period
